# Tetra­ammonium diaqua­diperoxidoocta­molybdate(VI) tetra­hydrate

**DOI:** 10.1107/S1600536809023460

**Published:** 2009-06-27

**Authors:** Antony J. Ward, Gregory J. Arrow, Thomas Maschmeyer, Anthony F. Masters, Peter Turner, Jack K. Clegg

**Affiliations:** a Laboratory of Advanced Catalysis for Sustainability, School of Chemistry, F11, The University of Sydney, Sydney, NSW 2006, Australia; b Crystal Structure Analysis Facility, School of Chemistry, F11, The University of Sydney, Sydney, NSW 2006, Australia

## Abstract

The title compound (NH_4_)_4_[Mo_8_O_24_(O_2_)_2_(H_2_O)_2_]·4H_2_O, consists of an octa­molybdate cluster with a crystallographic centre of symmetry. The clusters pack in a cubic close packing arrangement defining channels containing water mol­ecules and ammonium cations, which exhibit hydrogen bonding with neighbouring clusters. Hydrogen bonding also exists between the coordinated water mol­ecules of one cluster with one of the O atoms of the peroxido fragment in a neighbouring cluster.

## Related literature

For work on polyoxidomolybdates, see: Pope (1983 ▶); Pope & Müller (2001 ▶); Hill (1998 ▶). Baerwald (1885 ▶) probably reported the first peroxidomolybdate. Stomberg *et al.* have prepared a range of peroxidomolybdates and obtained crystal structures of these species, see: Larking & Stomberg (1970 ▶, 1972 ▶); Olson & Stomberg (1996 ▶, 1997*a* ▶,*b* ▶); Persdotter *et al.* (1986*a* ▶,*b* ▶,*c* ▶); Stomberg (1968 ▶, 1969 ▶, 1970 ▶, 1988*a* ▶,*b* ▶, 1992 ▶, 1995 ▶); Stomberg & Trysberg (1969 ▶); Stomberg & Olson (1996 ▶); Trysberg & Stomberg (1968 ▶, 1981 ▶). The versatile MoO_6_ octa­hedron building block [see: Pope & Müller (1991 ▶); Chen & Zubieta (1992 ▶)] results in an exceptionally large family of polyoxidomolybdates, see: Michailovski & Patzke (2006 ▶). For a review of the structural chemistry of peroxidomolybdates, see: Dickman & Pope (1994 ▶): Sergienko (2008 ▶). The tetra­ammonium salt of the centrosymmetric [Mo_8_O_24_(O_2_)_2_(H_2_O)_2_]^4−^ anion has been characterized with moderate precision, see: Trysberg & Stomberg (1981 ▶): Olson & Stomberg (1997*a* ▶). For bonds lengths in polyoxidomolybdates, see: Feng & Mao (2004 ▶); Long *et al.* (2003 ▶); Shi *et al.* (2006 ▶). 
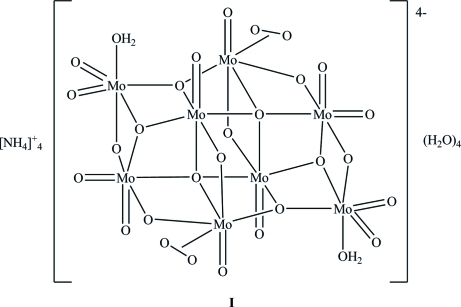


## Experimental

### 

#### Crystal data

(NH_4_)_4_[Mo_8_O_24_(O_2_)_2_(H_2_O)_2_]·4H_2_O
*M* *_r_* = 1395.78
Monoclinic, 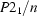

*a* = 10.405 (3) Å
*b* = 7.8706 (19) Å
*c* = 18.063 (4) Å
β = 96.991 (4)°
*V* = 1468.3 (6) Å^3^
*Z* = 2
Mo *K*α radiation
μ = 3.43 mm^−1^
*T* = 150 K
0.32 × 0.19 × 0.08 mm

#### Data collection

Bruker SMART 1000 CCD diffractometer
Absorption correction: gaussian (*XPREP*; Bruker, 1995 ▶; Coppens *et al.*, 1965 ▶) *T*_min_ = 0.398, *T*_max_ = 0.773
14005 measured reflections
3542 independent reflections
3434 reflections with *I* > 2σ(*I*)
*R*_int_ = 0.032

#### Refinement

*R*[*F* ^2^ > 2σ(*F* ^2^)] = 0.019
*wR*(*F* ^2^) = 0.043
*S* = 1.16
3542 reflections
250 parameters
14 restraints
Only H-atom coordinates refined
Δρ_max_ = 1.02 e Å^−3^
Δρ_min_ = −0.70 e Å^−3^

### 

Data collection: *SMART* (Bruker, 1995 ▶); cell refinement: *SAINT* (Bruker, 1995 ▶); data reduction: *SAINT* and *XPREP* (Bruker, 1995 ▶; Coppens *et al*., 1965 ▶); program(s) used to solve structure: *SIR97* (Altomare *et al.*, 1999 ▶); program(s) used to refine structure: *SHELXL97* (Sheldrick, 2008 ▶); molecular graphics: *TEXSAN for Windows* (Molecular Structure Corporation, 1998 ▶), *Xtal3*.7 (Hall *et al*., 2000 ▶), *ORTEPII* (Johnson, 1976 ▶) and *WinGX* (Farrugia, 1999 ▶); software used to prepare material for publication: *enCIFer* (Allen *et al.*, 2004 ▶).

## Supplementary Material

Crystal structure: contains datablocks global, I. DOI: 10.1107/S1600536809023460/br2108sup1.cif

Structure factors: contains datablocks I. DOI: 10.1107/S1600536809023460/br2108Isup2.hkl

Additional supplementary materials:  crystallographic information; 3D view; checkCIF report

## Figures and Tables

**Table 1:** Hydrogen-bond geometry (Å, °)

*D*—H⋯*A*	*D*—H	H⋯*A*	*D*⋯*A*	*D*—H⋯*A*
O12—H12*A*⋯O2^i^	0.947 (10)	1.802 (15)	2.715 (3)	161 (3)
O12—H12*A*⋯O1^i^	0.947 (10)	2.391 (16)	3.299 (3)	161 (3)
O12—H12*B*⋯O17^ii^	0.949 (10)	1.658 (12)	2.599 (3)	171 (4)
O16—H16*B*⋯O4^iii^	0.94 (3)	2.01 (3)	2.939 (3)	170 (3)
O16—H16*A*⋯O7	0.943 (10)	2.00 (2)	2.803 (3)	142 (3)
O17—H17*A*⋯O1	0.94 (3)	1.883 (16)	2.776 (3)	159 (3)
O17—H17*B*⋯O7^iv^	0.939 (10)	1.983 (12)	2.909 (3)	169 (3)
N1—H1*B*⋯O10^iv^	0.948 (10)	2.09 (3)	2.795 (3)	130 (3)
N1—H1*B*⋯O10^ii^	0.948 (10)	2.24 (3)	2.929 (3)	129 (3)
N1—H1*A*⋯O9^ii^	0.94 (3)	2.16 (2)	2.992 (3)	146 (3)
N1—H1*A*⋯O14	0.94 (3)	2.40 (3)	2.985 (3)	120 (3)
N1—H1*C*⋯O16^ii^	0.948 (10)	1.96 (2)	2.811 (3)	148 (3)
N1—H1*C*⋯O6^v^	0.948 (10)	2.36 (3)	3.038 (3)	128 (3)
N1—H1*D*⋯O17	0.95 (3)	2.17 (3)	2.859 (3)	129 (3)
N1—H1*D*⋯O8^v^	0.95 (3)	2.47 (3)	3.097 (3)	124 (3)
N1—H1*D*⋯O5	0.95 (3)	2.60 (3)	3.203 (3)	122 (3)
N2—H2*A*⋯O16	0.946 (10)	2.001 (14)	2.923 (3)	164 (3)
N2—H2*B*⋯O4	0.945 (10)	1.927 (14)	2.852 (3)	165 (3)
N2—H2*C*⋯O11^vi^	0.946 (10)	1.999 (15)	2.912 (3)	162 (3)
N2—H2*C*⋯O7^iii^	0.946 (10)	2.65 (3)	3.169 (3)	115 (3)
N2—H2*D*⋯O3^vii^	0.94 (3)	2.36 (3)	3.089 (3)	134 (3)
N2—H2*D*⋯O14^ii^	0.94 (3)	2.29 (3)	2.961 (3)	128 (3)

Symmetry codes: (i) 
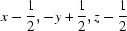
; (ii) 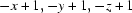; (iii) 
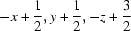
; (iv) 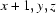; (v) 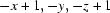; (vi) 
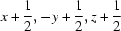
; (vii) 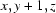.

**Fig. 1.**
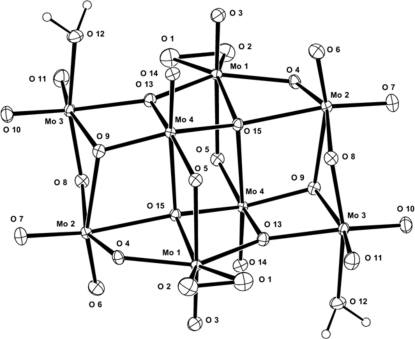
The molecular structure of the title complex, with displacement ellipsoids drawn at 50% probability level. Water solvent and ammonnium ions omitted for clarity. Symmetry code used for generating equivalent atoms: 1 - x, -y, 1 - z.

**Fig. 2.**
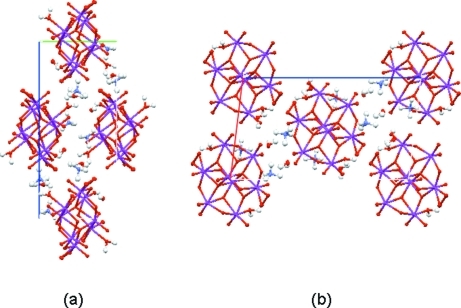
View along (a) the a axis and (b) the b axis of the crystal lattice of the title complex.

## supplementary crystallographic information

### Comment

Polyoxometalates, which constitute an enormous class of metal-oxygen cluster compounds, have become very widely utilized inorganic components due to the extreme variability of their composition, molecular characteristics and properties - see Pope (1983), Pope & Muller (2001), Hill (1998).

The aqueous chemistry of molybdenum is dominated by the formation of polyoxoanions, the key structural motif being the MoO_6_ octahedron - see Pope & Muller (1991), Chen & Zubieta (1992). This motif is a versatile building block that gives rise to an exceptionally large family of polyoxomolybdates which range from 3 to 368 metal ions in a single molecule - see Michailovski & Patzke (2006). Baerwald (1885) probably reported the first peroxomolybdate, the species resulting from the dissolution of ammonium paramolybdate in excess H_2_O_2_, which was formulated as 14NH_3_.18MoO_3_.3H_2_O_2_.18H_2_O, .

The structure of the title complex consists of an octamolybdate unit possessing an inversion centre (Figure 1). In the complex there is a peroxide ligand coordinated to Mo1, one water molecule bound to Mo3, two triply coordinated oxygen atoms, O9, O13, and one quadruply coordinated oxygen atom, O15. The Mo—O bond lengths with the polyvalent O atoms range from 2.0125 (18) to 2.3338 (19) Å. The bridging Mo—O bonds range in length length from 1.8753 (19) to 1.9753 (19) Å. The bond lengths for the terminal Mo=O bonds range from 1.686 (2) to 1.722 (2) Å. These bonds lengths are in good agreement with previously published polyoxomolybdate structures - see Long *et al.* (2003), Feng & Mao (2004), Shi *et al.* (2006). However, there are two bond lengths that show significant deviation from the expected: the Mo1—O5 bond length of 2.2836 (19) Å is extremely long for a bridging Mo—O bond while the Mo4—O9 bond length of 1.86089 (19) Å is considerably shorter than expected for a bond involving a triply bridging oxygen.

The packing of the title complex (Figure 2) shows the individual units to be stacked in a cubic close packing arrangement with water and ammonium ions distributed in the channels formed. Hydrogen bonding interactions exist between ammonium ions and the molybdenum cluster: H2B with O4, H1B with O10. In addition there exist hydrogen bonding interactions between the ammonium ions and the O atoms of neighbouring clusters: H2C with 011, H2D with O3, H1A with O9, and H1B with O10. The water molecules also hydrogen bond with the ammonium ions: O16 with H2A, O16 with H1C, and O17 with H1D. There is H-bonding between the H atoms of the water molecules with oxygen atoms of the molybdenum cluster: the strongest being that between O1 and H17A while 07 and H16A has a slightly longer hydrogen bond length. There also exists hydrogen bonding with the protons of the coordinated water molecules (H12A) of one cluster with the O2 atom in a neighbouring cluster while the other proton (H12B) has a strong hydrogen bond to 017. The water molecules also exhibit weak interactions with neighbouring clusters whereby H16A and H16B interact with O3 and H17A and H17B interact with O10.

### Experimental

Ammonium molybdate tetrahydrate (10 g, 8.1 mmol) was dissolved in a solution of hydrogen peroxide (30%, 50 ml) acidified to pH 2 with nitric acid (70%, 5 ml). Slow evaporation of the yellow solution afforded crystals of the title compound (7.45 g, 93%). Crystals suitable for XRD studies were obtained from an aqueous solution of the complex that was kept at 288 K and 80% humidity in order to reduce the rate of evaporation.

### Refinement

O-bound H atoms were located in the difference Fourier map and refined with bond length restraints of 0.95 (1) Å with *U*_iso_(H) 1.5 *U*_eq_(O).

### Crystal data

**Table:**

(NH_4_)_4_[Mo_8_O_24_(O2)_2_(H_2_O)_2_]·4H_2_O	*F*(000) = 1328
*M**_r_* = 1395.78	*D*_x_ = 3.157 Mg m^−^^3^
Monoclinic, *P*2_1_/*n*	Mo *K*α radiation, λ = 0.71073 Å
Hall symbol: -P 2yn	Cell parameters from 1023 reflections
*a* = 10.405 (3) Å	θ = 2.8–28.3°
*b* = 7.8706 (19) Å	µ = 3.43 mm^−^^1^
*c* = 18.063 (4) Å	*T* = 150 K
β = 96.991 (4)°	Blade, yellow
*V* = 1468.3 (6) Å^3^	0.32 × 0.19 × 0.08 mm
*Z* = 2	

### Data collection

**Table:**

Bruker SMART 1000 CCD diffractometer	3542 independent reflections
Radiation source: sealed tube	3434 reflections with *I* > 2σ(*I*)
graphite	*R*_int_ = 0.032
ω scans	θ_max_ = 28.3°, θ_min_ = 2.2°
Absorption correction: gaussian (*XPREP*; Bruker, 1995; Coppens *et al.*, 1965)	*h* = −13→13
*T*_min_ = 0.398, *T*_max_ = 0.773	*k* = −10→10
14005 measured reflections	*l* = −24→24

### Refinement

**Table:**

Refinement on *F*^2^	Primary atom site location: structure-invariant direct methods
Least-squares matrix: full	Secondary atom site location: difference Fourier map
*R*[*F*^2^ > 2σ(*F*^2^)] = 0.019	Hydrogen site location: inferred from neighbouring sites
*wR*(*F*^2^) = 0.043	Only H-atom coordinates refined
*S* = 1.16	*w* = 1/[σ^2^(*F*_o_^2^) + (0.016*P*)^2^ + 2.2853*P*] where *P* = (*F*_o_^2^ + 2*F*_c_^2^)/3
3542 reflections	(Δ/σ)_max_ = 0.001
250 parameters	Δρ_max_ = 1.01 e Å^−^^3^
14 restraints	Δρ_min_ = −0.70 e Å^−^^3^

### Special details

**Table:**

Experimental. attached with Exxon Paratone N, to a short length of fibre supported on a thin piece of copper wire inserted in a copper mounting pin. The crystal was quenched in a cold nitrogen gas stream from an Oxford Cryosystems Cryostream.
Geometry. All esds (except the esd in the dihedral angle between two l.s. planes) are estimated using the full covariance matrix. The cell esds are taken into account individually in the estimation of esds in distances, angles and torsion angles; correlations between esds in cell parameters are only used when they are defined by crystal symmetry. An approximate (isotropic) treatment of cell esds is used for estimating esds involving l.s. planes.
Refinement. Refinement of F^2^ against ALL reflections. The weighted R-factor wR and goodness of fit S are based on F^2^, conventional R-factors R are based on F, with F set to zero for negative F^2^. The threshold expression of F^2^ > 2sigma(F^2^) is used only for calculating R-factors(gt) etc. and is not relevant to the choice of reflections for refinement. R-factors based on F^2^ are statistically about twice as large as those based on F, and R- factors based on ALL data will be even larger.

### Fractional atomic coordinates and isotropic or equivalent isotropic displacement parameters (Å^2^)

**Table:**

	*x*	*y*	*z*	*U*_iso_*/*U*_eq_	
Mo1	0.578257 (18)	0.03860 (2)	0.654717 (10)	0.00883 (5)	
Mo2	0.267481 (18)	0.07806 (2)	0.612704 (10)	0.00964 (5)	
Mo3	0.153081 (18)	0.16838 (2)	0.442190 (11)	0.00992 (5)	
Mo4	0.473839 (18)	0.21902 (2)	0.486860 (10)	0.00802 (5)	
O1	0.75337 (18)	0.1060 (2)	0.69344 (10)	0.0214 (4)	
O2	0.65823 (18)	0.2109 (2)	0.72269 (10)	0.0216 (4)	
O3	0.54977 (16)	−0.1215 (2)	0.71260 (9)	0.0146 (3)	
O4	0.42192 (15)	0.1707 (2)	0.66940 (9)	0.0111 (3)	
O5	0.59440 (16)	0.2362 (2)	0.56376 (9)	0.0117 (3)	
O6	0.25417 (17)	−0.1077 (2)	0.65969 (9)	0.0153 (3)	
O7	0.15724 (16)	0.2132 (2)	0.64528 (10)	0.0154 (3)	
O8	0.17259 (16)	0.0052 (2)	0.51832 (9)	0.0123 (3)	
O9	0.32211 (15)	0.2731 (2)	0.52672 (9)	0.0112 (3)	
O10	0.06078 (16)	0.3204 (2)	0.47923 (10)	0.0154 (3)	
O11	0.05378 (17)	0.0689 (2)	0.37343 (10)	0.0178 (4)	
O12	0.20013 (18)	0.3722 (2)	0.36953 (10)	0.0165 (3)	
O13	0.32947 (15)	0.0976 (2)	0.41274 (9)	0.0108 (3)	
O14	0.50583 (16)	0.3819 (2)	0.42998 (9)	0.0133 (3)	
O15	0.55751 (15)	0.02784 (19)	0.43622 (9)	0.0095 (3)	
O16	0.1645 (2)	0.5543 (2)	0.69018 (11)	0.0235 (4)	
O17	0.89009 (18)	0.3251 (2)	0.61140 (10)	0.0181 (4)	
N1	0.7945 (2)	0.3830 (3)	0.45833 (12)	0.0163 (4)	
N2	0.4450 (3)	0.5128 (3)	0.72139 (13)	0.0225 (5)	
H12A	0.203 (3)	0.354 (5)	0.3179 (7)	0.034*	
H12B	0.162 (3)	0.481 (2)	0.372 (2)	0.034*	
H16B	0.128 (3)	0.597 (4)	0.7317 (13)	0.034*	
H16A	0.135 (3)	0.441 (2)	0.691 (2)	0.034*	
H17A	0.854 (3)	0.267 (4)	0.6492 (15)	0.034*	
H17B	0.9770 (14)	0.290 (4)	0.615 (2)	0.034*	
H1B	0.8775 (17)	0.434 (4)	0.465 (2)	0.034*	
H1A	0.728 (2)	0.462 (4)	0.463 (2)	0.034*	
H1C	0.797 (3)	0.361 (5)	0.4069 (7)	0.034*	
H1D	0.797 (4)	0.303 (4)	0.4976 (14)	0.034*	
H2A	0.3570 (14)	0.546 (5)	0.716 (2)	0.034*	
H2B	0.447 (4)	0.405 (2)	0.6981 (19)	0.034*	
H2C	0.468 (3)	0.505 (5)	0.7736 (7)	0.034*	
H2D	0.494 (3)	0.589 (4)	0.6960 (18)	0.034*	

### Atomic displacement parameters (Å^2^)

**Table:**

	*U*^11^	*U*^22^	*U*^33^	*U*^12^	*U*^13^	*U*^23^
Mo1	0.00872 (9)	0.00944 (9)	0.00819 (9)	0.00024 (6)	0.00051 (7)	−0.00073 (6)
Mo2	0.00888 (9)	0.01032 (9)	0.00991 (9)	−0.00013 (7)	0.00191 (7)	−0.00078 (7)
Mo3	0.00759 (9)	0.01116 (9)	0.01077 (9)	0.00088 (7)	0.00020 (7)	−0.00090 (7)
Mo4	0.00759 (9)	0.00779 (9)	0.00865 (9)	0.00016 (6)	0.00085 (7)	−0.00025 (6)
O1	0.0209 (9)	0.0228 (9)	0.0195 (9)	−0.0036 (8)	−0.0015 (7)	−0.0021 (7)
O2	0.0199 (9)	0.0253 (10)	0.0186 (9)	−0.0046 (8)	−0.0013 (7)	−0.0022 (7)
O3	0.0155 (8)	0.0143 (8)	0.0137 (8)	0.0014 (7)	0.0009 (6)	0.0009 (6)
O4	0.0108 (7)	0.0116 (7)	0.0111 (7)	0.0002 (6)	0.0016 (6)	−0.0033 (6)
O5	0.0115 (8)	0.0109 (7)	0.0126 (8)	−0.0006 (6)	0.0012 (6)	−0.0001 (6)
O6	0.0167 (8)	0.0146 (8)	0.0148 (8)	−0.0021 (7)	0.0030 (7)	−0.0002 (6)
O7	0.0119 (8)	0.0166 (8)	0.0183 (8)	0.0013 (6)	0.0045 (7)	−0.0031 (7)
O8	0.0120 (7)	0.0119 (7)	0.0129 (8)	−0.0017 (6)	0.0010 (6)	−0.0009 (6)
O9	0.0105 (7)	0.0099 (7)	0.0132 (8)	−0.0002 (6)	0.0010 (6)	−0.0007 (6)
O10	0.0108 (8)	0.0181 (8)	0.0177 (8)	0.0022 (7)	0.0029 (6)	−0.0019 (7)
O11	0.0145 (8)	0.0201 (9)	0.0177 (8)	0.0001 (7)	−0.0019 (7)	−0.0040 (7)
O12	0.0211 (9)	0.0143 (8)	0.0144 (8)	0.0035 (7)	0.0031 (7)	0.0034 (7)
O13	0.0093 (7)	0.0116 (7)	0.0113 (7)	−0.0001 (6)	0.0001 (6)	−0.0013 (6)
O14	0.0144 (8)	0.0123 (8)	0.0129 (8)	0.0003 (6)	0.0005 (6)	0.0008 (6)
O15	0.0090 (7)	0.0100 (7)	0.0093 (7)	0.0008 (6)	0.0008 (6)	−0.0012 (6)
O16	0.0344 (11)	0.0182 (9)	0.0186 (9)	−0.0031 (8)	0.0056 (8)	−0.0005 (7)
O17	0.0171 (9)	0.0166 (8)	0.0207 (9)	0.0037 (7)	0.0023 (7)	0.0026 (7)
N1	0.0146 (10)	0.0173 (10)	0.0166 (10)	−0.0010 (8)	−0.0004 (8)	−0.0002 (8)
N2	0.0322 (13)	0.0187 (11)	0.0159 (10)	−0.0009 (10)	0.0001 (9)	0.0021 (9)

### Geometric parameters (Å, °)

**Table:**

Mo1—O3	1.6864 (17)	Mo4—O15	2.0131 (16)
Mo1—O1	1.9443 (19)	Mo4—O13	2.1148 (16)
Mo1—O2	1.9468 (19)	Mo4—O15^i^	2.4335 (16)
Mo1—O13^i^	1.9599 (16)	O1—O2	1.438 (3)
Mo1—O4	1.9755 (16)	O12—H12A	0.947 (10)
Mo1—O15^i^	2.0983 (16)	O12—H12B	0.949 (10)
Mo1—O5	2.2836 (17)	O13—Mo1^i^	1.9599 (16)
Mo2—O6	1.7045 (18)	O15—Mo1^i^	2.0983 (16)
Mo2—O7	1.7200 (17)	O15—Mo2^i^	2.2768 (16)
Mo2—O4	1.9397 (16)	O15—Mo4^i^	2.4335 (16)
Mo2—O8	1.9496 (16)	O16—H16B	0.94 (3)
Mo2—O15^i^	2.2768 (16)	O16—H16A	0.943 (10)
Mo2—O9	2.3037 (17)	O17—H17A	0.94 (3)
Mo3—O11	1.7062 (18)	O17—H17B	0.939 (10)
Mo3—O10	1.7198 (17)	N1—H1B	0.948 (10)
Mo3—O8	1.8744 (17)	N1—H1A	0.94 (3)
Mo3—O13	2.0493 (17)	N1—H1C	0.948 (10)
Mo3—O12	2.1663 (18)	N1—H1D	0.95 (3)
Mo3—O9	2.3348 (16)	N2—H2A	0.946 (10)
Mo4—O14	1.7007 (17)	N2—H2B	0.945 (10)
Mo4—O5	1.7600 (16)	N2—H2C	0.946 (10)
Mo4—O9	1.8626 (17)	N2—H2D	0.94 (3)
			
O3—Mo1—O1	102.03 (8)	O12—Mo3—O9	85.81 (6)
O3—Mo1—O2	102.90 (8)	O14—Mo4—O5	104.22 (8)
O1—Mo1—O2	43.38 (8)	O14—Mo4—O9	107.38 (8)
O3—Mo1—O13^i^	96.49 (8)	O5—Mo4—O9	103.51 (8)
O1—Mo1—O13^i^	82.20 (8)	O14—Mo4—O15	99.30 (7)
O2—Mo1—O13^i^	124.68 (8)	O5—Mo4—O15	96.32 (7)
O3—Mo1—O4	95.75 (8)	O9—Mo4—O15	141.30 (7)
O1—Mo1—O4	123.99 (8)	O14—Mo4—O13	97.71 (7)
O2—Mo1—O4	81.05 (8)	O5—Mo4—O13	156.59 (7)
O13^i^—Mo1—O4	147.69 (7)	O9—Mo4—O13	77.16 (7)
O3—Mo1—O15^i^	98.44 (7)	O15—Mo4—O13	71.81 (6)
O1—Mo1—O15^i^	149.52 (7)	O14—Mo4—O15^i^	175.07 (7)
O2—Mo1—O15^i^	149.62 (7)	O5—Mo4—O15^i^	75.11 (7)
O13^i^—Mo1—O15^i^	73.21 (7)	O9—Mo4—O15^i^	77.47 (6)
O4—Mo1—O15^i^	75.48 (6)	O15—Mo4—O15^i^	76.00 (7)
O3—Mo1—O5	171.45 (7)	O13—Mo4—O15^i^	82.33 (6)
O1—Mo1—O5	85.71 (7)	O2—O1—Mo1	68.40 (11)
O2—Mo1—O5	85.18 (7)	O1—O2—Mo1	68.21 (11)
O13^i^—Mo1—O5	80.85 (7)	Mo2—O4—Mo1	111.97 (8)
O4—Mo1—O5	82.64 (6)	Mo4—O5—Mo1	114.05 (8)
O15^i^—Mo1—O5	73.02 (6)	Mo3—O8—Mo2	115.98 (8)
O6—Mo2—O7	105.20 (9)	Mo4—O9—Mo2	113.49 (7)
O6—Mo2—O4	99.89 (8)	Mo4—O9—Mo3	105.84 (7)
O7—Mo2—O4	97.53 (8)	Mo2—O9—Mo3	88.71 (6)
O6—Mo2—O8	96.89 (8)	Mo3—O12—H12A	121 (2)
O7—Mo2—O8	101.14 (8)	Mo3—O12—H12B	121 (2)
O4—Mo2—O8	150.60 (7)	H12A—O12—H12B	104 (3)
O6—Mo2—O15^i^	89.80 (7)	Mo1^i^—O13—Mo3	146.31 (9)
O7—Mo2—O15^i^	163.24 (7)	Mo1^i^—O13—Mo4	106.07 (7)
O4—Mo2—O15^i^	72.07 (6)	Mo3—O13—Mo4	107.61 (7)
O8—Mo2—O15^i^	84.07 (6)	Mo4—O15—Mo1^i^	104.76 (7)
O6—Mo2—O9	161.61 (7)	Mo4—O15—Mo2^i^	149.38 (8)
O7—Mo2—O9	92.75 (7)	Mo1^i^—O15—Mo2^i^	95.68 (6)
O4—Mo2—O9	81.31 (6)	Mo4—O15—Mo4^i^	104.00 (7)
O8—Mo2—O9	75.33 (6)	Mo1^i^—O15—Mo4^i^	97.11 (6)
O15^i^—Mo2—O9	73.00 (6)	Mo2^i^—O15—Mo4^i^	95.65 (6)
O11—Mo3—O10	106.56 (9)	H16B—O16—H16A	99 (3)
O11—Mo3—O8	102.80 (8)	H17A—O17—H17B	105 (3)
O10—Mo3—O8	101.94 (8)	H1B—N1—H1A	112 (3)
O11—Mo3—O13	99.70 (8)	H1B—N1—H1C	94 (3)
O10—Mo3—O13	148.24 (7)	H1A—N1—H1C	108 (3)
O8—Mo3—O13	89.08 (7)	H1B—N1—H1D	104 (3)
O11—Mo3—O12	93.46 (8)	H1A—N1—H1D	110 (3)
O10—Mo3—O12	84.18 (8)	H1C—N1—H1D	128 (3)
O8—Mo3—O12	159.99 (7)	H2A—N2—H2B	106 (3)
O13—Mo3—O12	76.61 (7)	H2A—N2—H2C	104 (3)
O11—Mo3—O9	168.36 (7)	H2B—N2—H2C	112 (3)
O10—Mo3—O9	84.95 (7)	H2A—N2—H2D	111 (3)
O8—Mo3—O9	75.90 (6)	H2B—N2—H2D	108 (3)
O13—Mo3—O9	68.81 (6)	H2C—N2—H2D	116 (3)

Symmetry codes: (i) −*x*+1, −*y*, −*z*+1.

### Hydrogen-bond geometry (Å, °)

**Table:**

*D*—H···*A*	*D*—H	H···*A*	*D*···*A*	*D*—H···*A*
O12—H12A···O2^ii^	0.95 (1)	1.80 (2)	2.715 (3)	161 (3)
O12—H12A···O1^ii^	0.95 (1)	2.39 (2)	3.299 (3)	161 (3)
O12—H12B···O17^iii^	0.95 (1)	1.66 (1)	2.599 (3)	171 (4)
O16—H16B···O4^iv^	0.94 (3)	2.01 (3)	2.939 (3)	170 (3)
O16—H16A···O7	0.94 (1)	2.00 (2)	2.803 (3)	142 (3)
O17—H17A···O1	0.94 (3)	1.88 (2)	2.776 (3)	159 (3)
O17—H17B···O7^v^	0.94 (1)	1.98 (1)	2.909 (3)	169 (3)
N1—H1B···O10^v^	0.95 (1)	2.09 (3)	2.795 (3)	130 (3)
N1—H1B···O10^iii^	0.95 (1)	2.24 (3)	2.929 (3)	129 (3)
N1—H1A···O9^iii^	0.94 (3)	2.16 (2)	2.992 (3)	146 (3)
N1—H1A···O14	0.94 (3)	2.40 (3)	2.985 (3)	120 (3)
N1—H1C···O16^iii^	0.95 (1)	1.96 (2)	2.811 (3)	148 (3)
N1—H1C···O6^i^	0.95 (1)	2.36 (3)	3.038 (3)	128 (3)
N1—H1D···O17	0.95 (3)	2.17 (3)	2.859 (3)	129 (3)
N1—H1D···O8^i^	0.95 (3)	2.47 (3)	3.097 (3)	124 (3)
N1—H1D···O5	0.95 (3)	2.60 (3)	3.203 (3)	122 (3)
N2—H2A···O16	0.95 (1)	2.00 (1)	2.923 (3)	164 (3)
N2—H2B···O4	0.95 (1)	1.93 (1)	2.852 (3)	165 (3)
N2—H2C···O11^vi^	0.95 (1)	2.00 (2)	2.912 (3)	162 (3)
N2—H2C···O7^iv^	0.95 (1)	2.65 (3)	3.169 (3)	115 (3)
N2—H2D···O3^vii^	0.94 (3)	2.36 (3)	3.089 (3)	134 (3)
N2—H2D···O14^iii^	0.94 (3)	2.29 (3)	2.961 (3)	128 (3)

Symmetry codes: (ii) *x*−1/2, −*y*+1/2, *z*−1/2; (iii) −*x*+1, −*y*+1, −*z*+1; (iv) −*x*+1/2, *y*+1/2, −*z*+3/2; (v) *x*+1, *y*, *z*; (i) −*x*+1, −*y*, −*z*+1; (vi) *x*+1/2, −*y*+1/2, *z*+1/2; (vii) *x*, *y*+1, *z*.
